# Recent standpoints on preoperative treatment of gastroenterological cancers: Who will be a real beneficiary?

**DOI:** 10.1002/ags3.12293

**Published:** 2019-10-22

**Authors:** Hideaki Shimada

**Affiliations:** ^1^ Department of Surgery and Clinical Oncology Toho University Graduate School of Medicine Tokyo Japan

Despite improvements in diagnostic tools, more than half of the patients with gastroenterological cancer still show metastases at the time of diagnosis. In patients without obvious distant metastases before surgery, rapid recurrence has been observed in certain subgroups. Such malignancy can be partially explained by “micro‐metastases” and/or “circulating tumor cells”.^1^ In the last 20 years, several randomized clinical trials have attempted to investigate the clinical efficacy of adjuvant chemotherapy.^2^ Postoperative adjuvant therapy has been confirmed as standard for esophageal, gastric, colorectal, and pancreatic cancers. Moreover, recent clinical trials have reported that preoperative treatment might be more favorable than postoperative treatment for esophageal cancer.

In recent articles, Pramesh et al reviewed the preoperative treatment of esophageal cancer,^3^ Komatsu et al reviewed clinical and basic topics in gastric cancer treatment,^1^ and Yamada et al reported on the prognostic impact of complete pathological response in pancreatic cancer.^4^ Recent well‐designed trials have established preoperative treatment strategies. Such trials have shown that these strategies produce superior clinical outcomes compared to surgery alone. However, optimum preoperative treatment strategy and appropriate real targets of specific subgroups have not been established. As Komatsu et al stated, circulating tumor DNAs and/or RNAs may be useful tools in selecting precise high‐risk groups before surgery.^1^

Appropriate criteria required to identify the “real responder” and/or “real beneficiary” have not been established in pancreatic cancer. Yamada et al partially addressed this question in patients with pancreatic cancer. They concluded that the complete pathological response, from a prognostic standpoint, should be defined as the complete absence of an invasive component regardless of the residual intraductal carcinoma component.^4^

“Who will be the real beneficiary of preoperative treatment?” should be one of the hot topics at the moment.

## DISCLOSURE

Conflicts of Interest: Author declares no conflicts of interest for this article.
